# Liu Wei formula ameliorates EGFR-induced malignant glioma progression by regulating EGFR/PI3K/AKT pathway and T-cell antitumor immunity

**DOI:** 10.3389/fphar.2026.1825282

**Published:** 2026-07-13

**Authors:** Yan Li, Tingting Hu, Wenhao Xu, Ruming Huang, Fanrui Kong, Yuyan She, Yubei Xia, Yajunyu Wu, Songru Guo, Peng Cao, Ning Wang

**Affiliations:** 1 School of Acupuncture-Moxibustion and Tuina of Nanjing University of Chinese Medicine, Nanjing, China; 2 State Key Laboratory of Technologies for Chinese Medicine Pharmaceutical Process Control and Intelligent Manufacture, Nanjing University of Chinese Medicine, Nanjing, China; 3 Zhongda Hospital Southeast University Jiangbei (Nanjing Dachang Hospital), Nanjing, China; 4 Taikang Xianlin Drum Tower Hospital, Nanjing, Jiangsu, China

**Keywords:** baicalin and luteolin, epidermal growth factor receptor/phosphatidylinositol-3-kinase/protein kinase B pathway, EGFR-induced glioma, Liu Wei formula, network pharmacology, T-cell infiltration

## Abstract

**Background:**

Gliomas, common malignant intracranial tumors, are associated with a poor prognosis and pose a significant threat to human health. Liu Wei formula (LWF), a century-old herbal formula developed by Tai Kang Gu Lou Hospital of Nanjing University of Chinese Medicine, shows clinical efficacy against glioma. However, the fundamental molecular mechanisms and pathways through which LWF exerts its therapeutic effects remain largely unclear.

**Materials and Methods:**

A LUC-GL261 mouse model was established to assess the *in vivo* efficacy of LWF. Network pharmacology analysis was used to identify target enrichment regions between active components of LWF and glioma cells. Western blot (WB) was performed to evaluate the impact of LWF on human EGFR, phosphatidylinositol 3-kinase (PI3K), P-PI3K, protein kinase B (AKT), and P-AKT expression. Network pharmacology and differential gene analysis were used to identify differentially expressed genes and key signaling pathways, and quantitative real-time PCR (qPCR) was used to validate changes in target genes in EGFR-related pathways affected by the administration of LWF. Flow cytometry was used to detect changes in the proportion of T cells in tumor lymph nodes of mice treated with LWF. Surface plasmon resonance (SPR), molecular docking, and WB were performed to explore the functional ingredients of LWF. Additional clinical research is needed to clarify the clinical efficacy of LWF.

**Results:**

LWF significantly suppressed tumor growth in mice. Network pharmacology and experimental validation revealed inhibition of the EGFR/PI3K/AKT pathway and downregulation of related genes. Flow cytometry analysis revealed a significant increase in the proportion of CD8^+^T cells in mouse lymph nodes after LWF treatment. Baicalin and luteolin, as active ingredients of LWF, play important roles in the treatment of glioma. LWF demonstrated durable antitumor activity in glioma patients and improved clinical symptoms, including dizziness, headache, and nausea.

**Conclusion:**

The above results indicate that LWF, through its main antitumor components baicalin and luteolin, suppresses malignant progression of EGFR-induced glioma by blocking the EGFR/PI3K/AKT pathway and enhancing T-cell expansion and function. This study offers a dual contribution: it presents clinical candidates for EGFR-driven glioma therapy and introduces a novel framework for determining the functional basis of LWFs.

## Graphical Abstract

**Figure:**
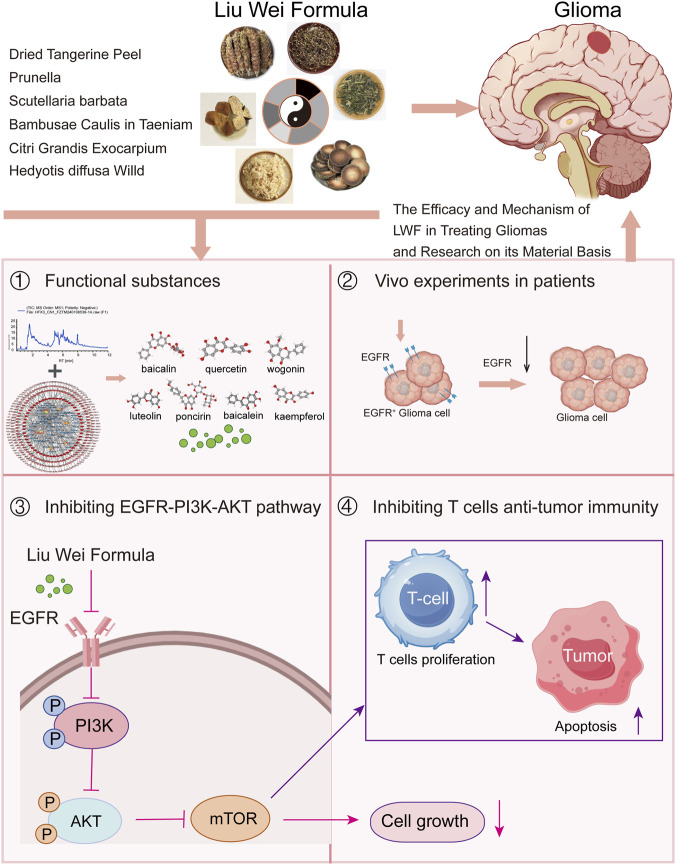

## 1 Introduction

Glioma is a clinically common type of malignant intracranial tumor (Weller et al., 2024). According to statistics, the incidence rate of glioma in China ranges from 5 to 8 per 100,000 people, with its five-year mortality rate ranking third among systemic tumors (Yang et al., 2022). The median overall survival is only 14–17 months. Infiltrative growth of the tumor within the brain can lead to increased intracranial pressure and neuropsychiatric changes, manifesting as clinical symptoms such as headaches, diplopia, nausea, and seizures. Clinical diagnosis primarily relies on pathological examination (Lan et al., 2024). The main current treatment approach involves surgical resection of the localized mass combined with radiotherapy and chemotherapy.

However, due to its predominantly infiltrative growth pattern and the intricate network of intracranial blood vessels and neural tissues, the boundary between normal and pathological brain tissue is often indistinct, making complete resection challenging (Czarnywojtek et al., 2023a). Following radiotherapy and chemotherapy, most patients experience adverse reactions such as fatigue, nausea, vomiting, and decreased liver function. Moreover, due to the blood–brain barrier, even targeted drug therapies have shown unsatisfactory efficacy in clinical trials (Czarnywojtek et al., 2023a; Czarnywojtek et al., 2023b). The poor prognosis and short survival period of glioma patients remain significant challenges in clinical diagnosis and treatment.

Current research indicates that the amplification of EGFR is a common feature in adult high-grade glioma (Romo et al., 2023). Studies have found widespread abnormal amplification and overexpression of EGFR in tumor patients. On the one hand, EGFR gene amplification typically leads to elevated protein expression levels, activating downstream RAS/RAF/MAPK, PI3K/AKT/mTOR, and STAT signaling pathways, thereby promoting tumor cell proliferation and metastasis (Singh et al., 2025; Hasan et al., 2024). On the other hand, pathological products of tumors can also activate the MAPK signaling cascade (Li et al., 2021).

Currently, EGFR amplification and mutation account for more than 50% of genetic alterations in glioma (Brennan et al., 2013; Okamoto et al., 2024). EGFR is a well-established clinical marker in glioma, frequently associated with an adverse prognosis. The EGFR signaling pathway is a key driver of tumorigenesis, promoting uncontrolled proliferation and survival of glioma cells (Chen et al., 2023; Saadeh et al., 2018). Evidence indicates that EGFR acts as a central regulator, impacting multiple signaling pathways critical to glioma pathogenesis (Fang et al., 2021; Liu et al., 2021). Aberrant EGFR signaling was associated with a unique tumor immune microenvironment in a study that emphasized that EGFR alterations were associated with significantly diminished infiltration of cytotoxic T cells (Xu et al., 2021). Simultaneously, it is a key molecule that promotes the malignant progression of glioma through multiple factors. Tumor-associated monocytes promote mesenchymal transformation through EGFR signaling in glioma (Chen et al., 2023).

Consequently, targeting EGFR represents a critical therapeutic strategy to halt the malignant progression of glioma. Clinically, EGFR-targeted drugs mainly fall into two categories: monoclonal antibodies that block activation of the tyrosine kinase domain and tyrosine kinase inhibitors (TKIs) designed to competitively bind to ATP sites. Both EGFR-targeted drugs aim to interrupt related signaling pathways. However, clinical studies have issues such as drug resistance and significant toxic side effects (Habibi et al., 2025). Further research is needed to develop more potent drugs and optimize combination strategies to improve outcomes.

In recent years, with deepening cancer research and growing emphasis on the “preventive treatment” theory, TCM has gained recognition for its active components (Li S. et al., 2025; Zhang et al., 2021). TCM shows promise in cancer prevention and treatment. Its holistic regulatory philosophy, characterized by multi-component, multi-target, and multi-pathway interventions, offers a novel paradigm for glioblastoma treatment. The components of the formula exhibit characteristics such as reducing toxicity, enhancing efficacy, minimizing adverse reactions, and acting on multiple targets (Xia et al., 2022).

Liu Wei formula (LWF), composed of Citrus reticulata Blanco (15 parts), Prunella vulgaris L. (20 parts), Scutellaria barbata D.Don (20 parts), Bambusa beecheyana Munro (12 parts), Citrus grandis (L.) Osbeck (15 parts), and Scleromitrion diffusum (Willd.) R.J.Wang (20 parts), is an effective prescription for treating glioma (details are presented in Table 1). The plant names were recorded in the Pharmacopeia of the People’s Republic of China and have been checked with MPNS (http://mpns.kew.org), accessed on 6 November 2025.

**TABLE 1:** Liu Wei formula.

Herb	Latin name	Family	Part used	Chinese name
Citri Reticulatae pericarpium	Citrus reticulata Blanco	Rutaceae	Fruit exocarp Fruit mesocarp	Chen Pi
Prunellaespica	Prunella vulgaris L.	Labiatae	Spike	Xia Ku Cao
Scutellariae barbatae herba	Scutellaria barbata D.Don	Labiatae	Dried herb	Ban Zhi Lian
Bambusae caulis in Taenias	Bambusa tuldoides Munro	Gramineae	Dried middle shavings of stem	Zhu Ru
Hedyotis diffusa Willd	Scleromitrion diffusum (Willd.) R. J. Wang	Rubiaceae	Dried herb	Bai Hua She She Cao
Citri grandis exocarpium	Citrus grandis (L.) Osbeck	Rutaceae	Dried exocarp of unripe or almost ripe fruit	Hua Ju Hong

Originating from the Wang family’s ancestral theory on cancer treatment and refined through decades of clinical practice, this formula has been successfully applied in the treatment of various tumors at Nanjing Taikang Gulou Hospital. This study explores its role and mechanism in glioma. It is worth noting that LWF has demonstrated anti-glioma effects in both clinical and animal studies. Meanwhile, it improves symptoms such as headache and nausea. As the core patented formula of the Wang family’s cancer treatment system, all other cancer-treating prescriptions are modified versions derived from this foundational formula. The key components in the prescription, such as dried tangerine peel, Prunella vulgaris L., Scleromitrion diffusum (Willd.) R.J.Wang, and Scutellaria barbata D.Don, are heat-clearing and detoxifying herbs that can effectively inhibit tumor cell proliferation, invasion, and migration (Wang et al., 2023; Kim et al., 2020; Han et al., 2020; Sun et al., 2024). It represents the core technology and essence of the Wang family’s therapeutic approach. With precise ingredient compatibility and meticulous selection of herbs, refined over generations, it has consistently shown excellent clinical outcomes.

The emergence of network pharmacology has provided a critical tool for deciphering the systemic mechanisms of such TCM formulations. This study leverages network pharmacology and molecular docking techniques to investigate the pharmacodynamic effects and mechanisms by which LWF suppresses the malignant progression of glioma through EGFR inhibition. This work will investigate the specific mechanisms by which LWF inhibits glioblastoma, based on pharmacological evidence from clinical and animal studies.

## 2 Materials and methods

### 2.1 Cell lines, reagents, and compounds

GL261 glioma cells were cultured using DMEM containing 10% serum. Bevacizumab was purchased from Tamo Jiangsu Province Hospital on Integration of Chinese and Western Medicine. LWF was provided by Wang’s Lab. Baicalin (CAS: 21967-41-9) and luteolin (CAS: 491-70-3) were purchased from Target Mol.

### 2.2 Glioma xenograft assay

GL261 cells were washed with PBS, digested with trypsin, centrifuged, and resuspended in PBS to a final concentration of 6 × 10^7^ cells/mL. The cell suspension was kept on ice. Forty nude mice, 5 weeks old, were acclimatized for 1 week. GL261 cells were subcutaneously injected into the mice. Once tumor volumes reached 100 mm^3^, the mice were divided into six groups: vehicle group, bevacizumab treatment group, low-dose LWF group, high-dose LWF group, low-dose LWF combined with bevacizumab group, and high-dose LWF combined with the bevacizumab group. Mouse body weights and tumor volumes were recorded every other day for 21 days. The research was conducted in accordance with the internationally accepted principles for laboratory animal use and care as found in the U.S. guidelines (NIH publication #85-23, revised in 1985). The ethics permit number for the use of animals is SYXK (苏) 2021-0025. The ethics approval of the animals is AEWC-20220329-198.

### 2.3 Clinical case report analysis

EGFR-induced glioma patients were recruited from Taikang Drum Tower Hospital. Their medical histories, symptoms, and pre-/post-treatment brain MRI tumor size changes were analyzed to (1) evaluate LWF’s therapeutic efficacy against glioma and (2) assess its role in improving glioma-related complications. The research followed the guidelines of the Declaration of Helsinki and Tokyo for humans and was approved by the institutional human experimentation committee. Informed consent was obtained. This clinical study has been approved by the Medical Ethics Committee of Taikang Xianlin Gulou Hospital, reference number AF/SC-08/01.0, which is provided in the attachment. Clinical trial number: not applicable. The consent to participate declarations: not applicable.

### 2.4 Western blot (WB)

Cell proteins were extracted using RIPA lysis buffer. The specific experimental procedures were performed with primary antibodies, including EGFR (CSB-PA005571LA01HU) obtained from CUSABIO. PI3K (60225-1-1g) and AKT (10176-2-AP) antibodies were purchased from Protein Tech, and P-PI3K(17366s) and P-AKT (4060s) antibodies were obtained from Cell Signaling Technology.

### 2.5 Qualitative and quantitative analysis of metabolites

Preliminary screening is performed based on parameters such as retention time (RT) and mass-to-charge ratio (m/z). Peak alignment is conducted across different samples to enhance identification accuracy. Subsequently, peak extraction is carried out using predefined information including ppm tolerance and adduct ions, while simultaneously quantifying peak areas. Metabolite identification is then achieved by searching against high-resolution MS/MS spectral databases (mzCloud and mzVault) and the MassList primary mass database.

### 2.6 Metabolite quantification results

Using Compound Discoverer 3.3 (CD3.3) data processing software, chromatographic peaks detected in the samples were integrated. The peak area of each feature peak represents the relative quantitative value (relative abundance) of a metabolite. The quantitative results were normalized using the total peak area, ultimately yielding the metabolite quantification results.

### 2.7 Flow cytometry

The tumor lymph nodes were separated, and the tissue was ground to obtain a single-cell suspension. Flow cytometry labeling and staining were performed using CD45, CD3, CD4, and CD8 antibodies, respectively. The stained cell suspension was detected in a flow cytometer, and the proportions of CD3^+^CD8^+^T cells and CD3^+^CD4^+^T cells were counted. Antibodies targeting human proteins were employed in flow cytometry: CD45 (Biolegend, 103114), CD3 (Biolegend, 108412), CD4 (eBioscience 17-0042-81), and CD8 (Biolegend, 100708).

### 2.8 Statistical analysis

Statistical software GraphPad Prism 9.0 was used to analyze and process the obtained data. The differences between the two groups were analyzed using the Student t-test, whereas the differences between multiple groups were analyzed using one-way analysis of variance. The results were expressed as the mean ± standard error of the mean. A *p-*value <0.05 indicated a significant difference. Statistical data are provided in Supplementary Materials.

## 3 Results

### 3.1 Chemical components identified in LWF

**FIGURE 1:**
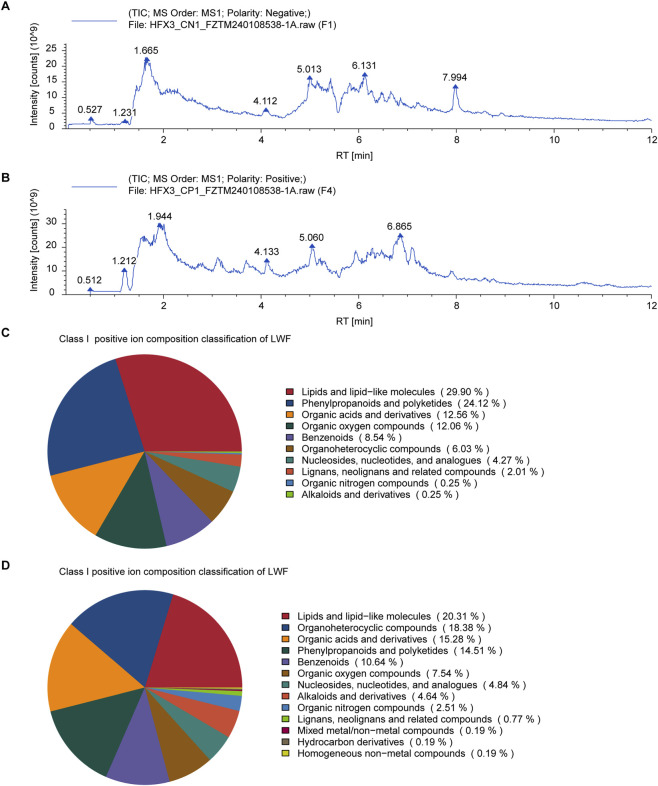
Analysis of the chemical composition of LWF. LWF total ion chromatogram (TIC): **(A)** positive ion mode, **(B)** negative ion mode, **(C)** negative ion composition classification of LWF, and **(D)** positive ion composition classification of LWF.

First, we prepared freeze-dried powder of LWF and characterized its chemical composition using the UHPLC-Q Exactive system. Figures 1A,B show the total ion chromatograms (TIC) of LWF in positive and negative ion modes, respectively. Baseline filtering and peak identification were performed on the original data using the Compound Discoverer 3.3 (CD3.3) data processing software to obtain the data matrix, and the characteristic peaks were searched, identified, and matched. The MS quality error was set to be < 5 ppm. According to the matching score of secondary ion mass spectrometry, 1,209 chemical components of LWF were identified. The chemical components of LWF were divided into 13 categories, including lipids, organoheterocyclic compounds, organic acids, organic oxygen compounds, nucleosides, alkaloids, lignans, and so on (Figures 1C,D).

### 3.2 LWF suppresses the growth of LUC-GL261 xenografts in mice

**FIGURE 2:**
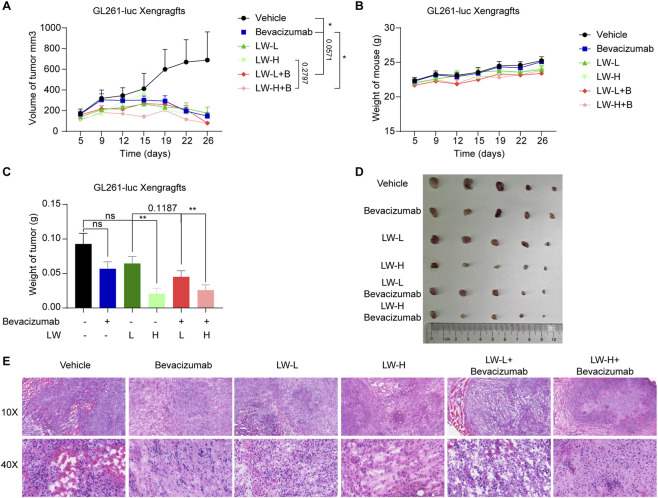
LWF suppresses the growth of LUC-GL261 xenografts in mice. **(A)** Tumor volume of all the LUC-GL261 xenograft treatment groups: vehicle group, bevacizumab group, low-dose LWF group, high-dose LWF group, low-dose LWF combined with the bevacizumab group, and high-dose LWF combined with the bevacizumab group; **(B)** body weight changes of mice across different treatment groups during the experimental period. **(C)** Comparative analysis of tumor mass among the experimental groups. **(D)** Representative images of glioma tissues resected from mice in each group. **(E)** H&E staining of glioma tissues across groups. Results are expressed as the mean ± SEM, N = 5; *p < 0.05, **p < 0.01. n.s., not significant.

**FIGURE 3:**
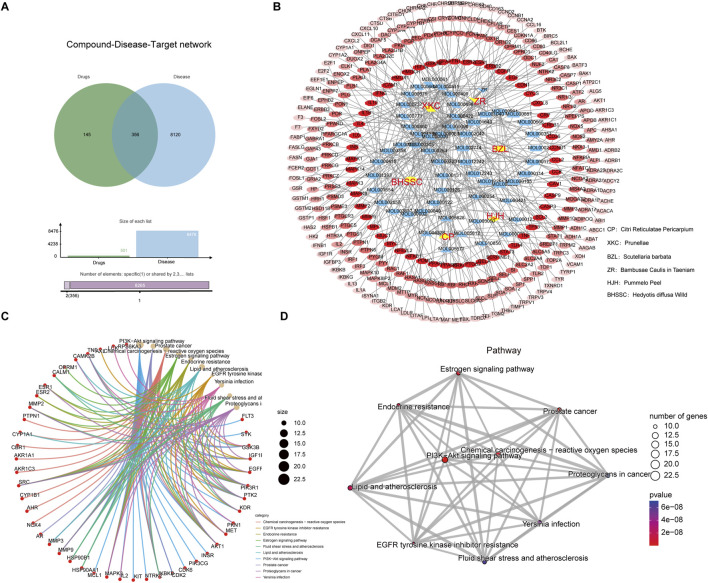
Exploring the relationship between LWF and glioma targets through network pharmacology. **(A)** Venn diagram analysis of LWF targets and glioma targets. **(B)** Network of drug–component–targets: active ingredient drug target network diagram of LWF. **(C)** Signal network diagram of potential targets and pathways. **(D)** Connection between key signaling pathways.

To validate LWF’s inhibition of glioma progression, we established a xenograft Luc-GL261 glioma mouse model. Tumor volume and body weight were monitored for 21 days. As shown in Figure 3A, LWF exhibited significant antitumor activity in Luc-GL261 xenografts, as evidenced by the repressed tumor volume and tumor weight. According to the anatomical diagram of the tumor, compared with the low-dose group of LWF, the high-dose group of LWF can significantly inhibit the growth of glioma. What is even more surprising is that the combination of low-dose LWF and bevacizumab has a significantly better effect on inhibiting glioma than using low-dose LWF alone (Figures 2A,C,D). Meanwhile, no change in the mice’s body weight was observed following administration (Figure 3B). Hematoxylin and eosin (H&E) staining of tumor tissues revealed significantly reduced cell counts and markedly decreased cellular density in the high-dose LWF and combination therapy group (Figure 3E). To sum up, LWF can inhibit the malignant progression of glioma in mice.

### 3.3 Network pharmacology analysis of LWF targets with glioma

To explore the specific action mechanism of LWF, we conducted analysis through network pharmacology and a Venn diagram to interactively compare the standardized disease-related targets with TCM-related targets. The SwissTargetPrediction database and Target Net database were used to predict the compound targets.

As shown in Figure 3A, 356 targets were enriched between LWF targets and glioma targets (Figures 3A,B). Using drug–component–targets network analysis, we built an interaction between the six traditional Chinese herbs in LWF with different targets (Figure 3B), highlighting the advantages of multi-component and multi-target TCM. By enriching signal pathways through key targets, a network of intersecting targets and signal pathways is established (Figure 3C). At the same time, an interlocking network between signal pathways was constructed (Figure 3D), which demonstrated that PI3K-AKT and EGFR tyrosine kinase inhibitor (TKI)-resistant pathways were significantly enriched. To sum up, LWF can prominently affect multiple signaling pathways related to glioma growth through various components, especially proliferation-related signaling pathways such as EGFR/PI3K/AKT.

### 3.4 LWF inhibits malignant progression of glioma by suppressing EGFR expression and the PI3K-AKT pathway

**FIGURE 4:**
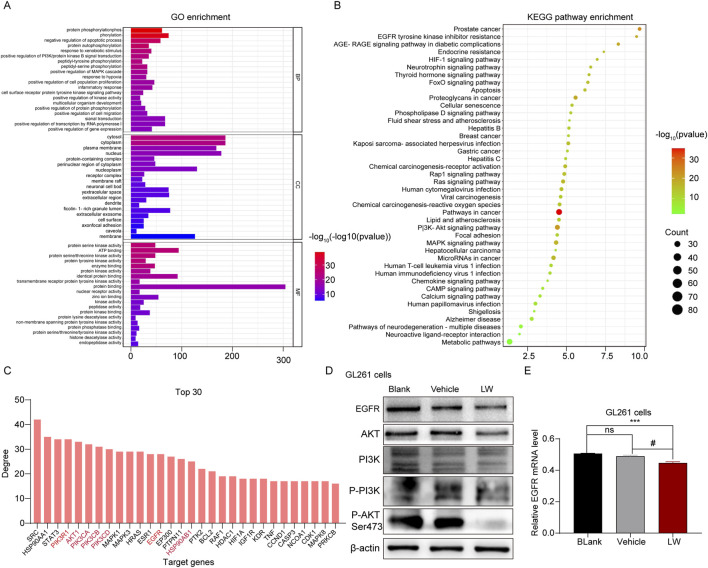
LWF inhibits malignant progression of glioblastoma by suppressing EGFR expression. **(A)** GO analysis diagram of common targets between LWF and glioma. **(B)** KEGG pathway analysis diagram of common targets between LWF and glioma. **(C)** The top 30 targets in the interaction score between LWF and diseases. **(D)** EGFR, AKT, P-AKT, PI3K, and P-PI3K protein expression in GL261 cells with the administration of the blank, blank serum, and serum containing LWF for 48 h. **(E)** The mRNA levels of EGFR with treatment containing LWF serum for 24 h.

To determine the specific pathways and targets of LWF, we identified the top 10 signaling pathways with the most significant correlation based on Kyoto Encyclopedia of Genes and Genomes (KEGG) and Gene Ontology (GO) enrichment analysis results, and labeled the targets involved in key signaling pathways. As shown in Figures 4A,B, the EGFR/PI3K-AKT signaling pathways are closely related to the action of LWF, and the PI3K-AKT signaling pathway is closely related to EGFR. Through further data analysis, the top 30 targets in the interaction score between LWF and diseases were selected, which were closely related to the EGFR/PI3K/AKT signaling pathway (Figure 4C).

The above results indicate that LWF-containing serum reduces the protein expression and phosphorylation levels of EGFR, PI3K, and AKT in GL261 cells, while simultaneously downregulating EGFR mRNA levels. These findings suggest that LWF exerts an inhibitory effect on the EGFR-PI3K-AKT pathway. However, it should be noted that the current evidence is primarily based on observations at the protein expression level and does not yet directly demonstrate functional inhibition of this pathway. Further validation through pathway rescue experiments is required.

To validate LWF’s modulation of the EGFR/PI3K/AKT signaling pathway, we detected the expression of EGFR, PI3K, P-PI3K, AKT, and p-AKT proteins through WB and PCR. As shown in Figures 4D,E, the serum of LWF can significantly inhibit EGFR, PI3K, P-PI3K, AKT, and p-AKT protein expression, and, to some extent, suppress EGFR mRNA levels. Based on these results, we concluded that LWF inhibited the malignant progression of glioma by suppressing the EGFR/PI3K/AKT pathway.

### 3.5 LWF enhances CD8^+^ T-cell infiltration in tumor lymph nodes

**FIGURE 5:**
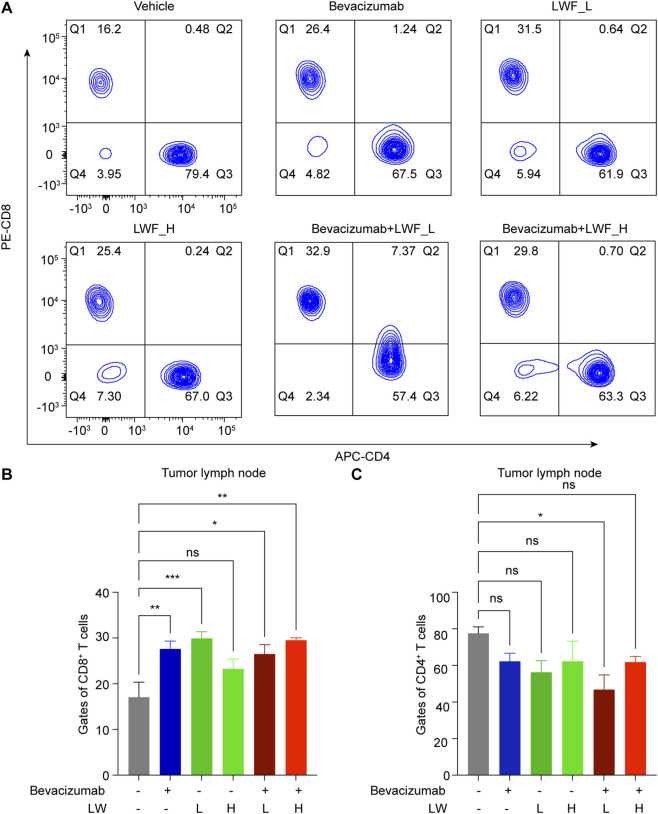
LWF increases CD8^+^ T-cell infiltration in tumor lymph nodes. **(A)** The CD8^+^ and CD4^+^ T cell gates in tumor lymph nodes. **(B)** The gates of CD8^+^ T cells. **(C)** The gates of CD4^+^ T cells. Results are expressed as mean ± SEM, N = 5; *p < 0.05, **p < 0.01. ***p < 0.001. n.s., not significant.

To investigate the effect of LWF on the immunity of glioma mice, especially the role of CD8^+^T-cell infiltration, we collected tumor lymph nodes from mice after the administration of LWF or LWF and bevacizumab. Flow cytometry labeling and staining were performed using CD45, CD3, CD4, and CD8 antibodies, respectively. The experimental results showed that LWF and LWF combined with bevacizumab can significantly increase the proportion of CD8^+^T cells in lymph nodes (Figures 5A,B). However, the proportion of CD4^+^T cells decreased (Figures 5A,C). The above results indicate that LWF can increase the infiltration of CD8^+^T cells and cause changes in the immune microenvironment.

### 3.6 Baicalin and luteolin in LWF suppress glioma by binding EGFR

**FIGURE 6:**
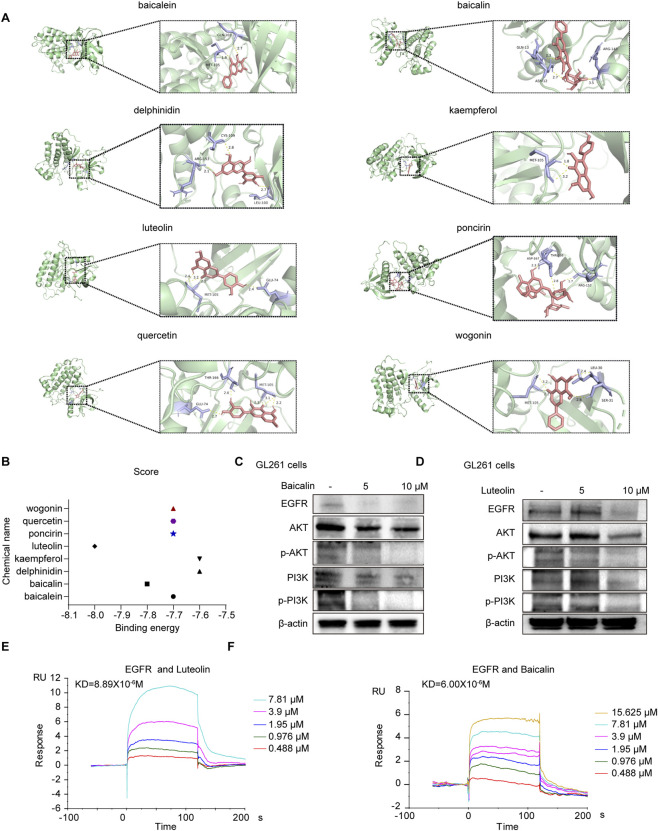
Baicalin and luteolin in LWF suppress glioblastoma by binding EGFR. **(A)** The docking between LWF chemicals and EGFR. **(B)** The docking scores of LWF chemicals and EGFR. **(C)** The protein expression levels of EGFR, P-PI3K, PI3K, P-AKT, and AKT with the administration of baicalin in GL261 cells. **(D)** The protein expression levels of EGFR, P-PI3K, PI3K, P-AKT, and AKT with the administration of luteolin in GL261 cells. **(E)** SPR of EGFR and baicalin. **(F)** SPR of EGFR and luteolin.

Building on EGFR’s critical role in glioma pathogenesis and the inhibitory effect on EGFR, we investigated the specific active components of LWF responsible for modulating EGFR. Initial molecular docking comprehensively evaluated specific compounds in the LWF capable of binding to EGFR. Results demonstrated that wogonin, quercetin, poncirin, luteolin, kaempferol, delphinidin, baicalin, and baicalein exhibited binding energies < −7 kcal/mol and precisely identified key binding sites at specific amino acid residues of EGFR. Notably, both luteolin and baicalin exhibited significantly stronger binding affinity to EGFR, with binding energies of −8.0 kcal/mol and −7.8 kcal/mol, respectively (Figures 6A,B). The extracted ion chromatogram (XIC) of part molecular is shown in Supplementary Figures S1A-D.

To elucidate the roles of baicalin and luteolin in targeting EGFR and inhibiting the EGFR/PI3K/AKT signaling pathway, we examined their effects on protein expression. The results demonstrated that both compounds dose-dependently suppressed the expression of EGFR, PI3K, P-PI3K, AKT, and P-AKT (Figures 6E,F). To directly confirm EGFR binding by luteolin and baicalin, we performed SPR assays. The binding kinetics revealed dissociation constants KD of 6 × 10^−6^ M for baicalin–EGFR and 88.89 × 10^−6^ M for luteolin–EGFR (Figures 6C,D). Collectively, these findings indicate that baicalin and luteolin in LWF are the principal active constituents mediating EGFR suppression and the observed anti-glioma efficacy.

### 3.7 LWF can significantly inhibit glioma growth in clinical cases

To validate the clinical efficacy of LWF in EGFR-positive glioma patients, we conducted genetic testing to identify patients with EGFR amplification and evaluate the therapeutic effect of LWF. Based on next‐generation sequencing technology, next‐generation sequencing (NGS) was conducted on a patient tumor sample and peripheral blood to detect four types of genetic variations in 889 genes associated with tumor development, including point mutations, small fragment insertions, deletions (Indels), copy number variations (CNVs), and currently known fusion genes.

**FIGURE 7:**
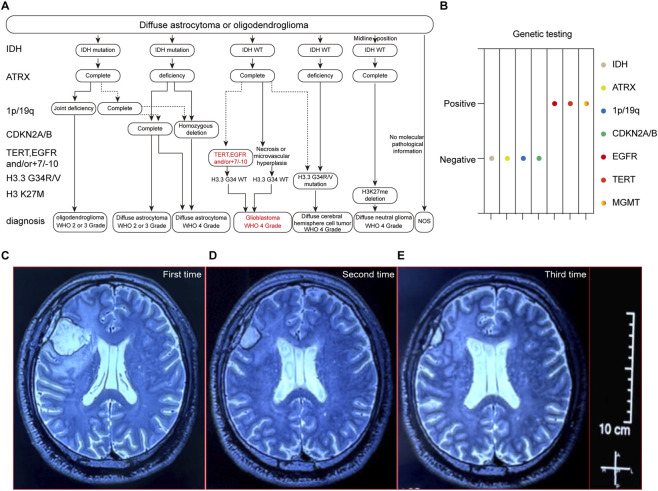
Clinical efficacy of LWF in glioma patients with high EGFR expression. **(A)** The Diagnosis and Treatment Guidelines for Gliomas (2022 Edition). **(B)** Genetic testing results. **(C)** Brain MRI images of the patient before treatment with LWF. **(D)** Brain MRI images of the patient after 2 months of LWF treatment. **(E)** Brain MRI images of the patient after 4 months of LWF treatment. The three magnetic resonance imaging test result maps are on the same scale.

The results of the gene analysis are as follows: according to the Diagnosis and Treatment Guidelines for Gliomas (2022 Edition), the patient had a high-grade glioma, histological equivalent to CNS WHO grade 4 or above (Figure 7A). The whole-gene testing of the patient showed negative IDH1/IDH2, negative 1p19q, positive TERT C288T, an average MGMT methylation level of 65%, and amplification of EGFR (Figure 7B). The head MRI plain scan and enhanced magnetic resonance imaging of the patient showed circular enhancement of the right frontal margin and thickening enhancement of the adjacent dura mater (Figure 7C).

Following treatment with the LWF, the patient exhibited a significant reduction in tumor burden. The head MRI plain scan and enhancement showed a reduced range compared to the previous film and only showed a small amount of glial hyperplasia around it (Figure 7D). Three months later, the tumor had transformed from malignant to cystic lesions with a significant reduction in tumor size (Figure 7E). In summary, after several months of LWF treatment, the patient exhibited significant tumor shrinkage, which demonstrated that LWF could inhibit the growth of glioma in patients.

Notably, sustained treatment with LWF achieved effective disease control of Liu’s glioma, demonstrating significant clinical stability. After 4 months of treatment with LWF, the patient’s head MRI revealed a benign cystic lesion (Supplementary Figure S2A). Based on these preliminary clinical observations, LWF appears to offer potential benefits in the treatment of malignant gliomas. After 10 months of treatment with LWF, the patient’s unclear speech improved, and they were able to walk independently, with resolution of dizziness (Supplementary Figures S2B-E). Tumor-related neurological symptoms (headaches and dizziness) were alleviated, likely due to tumor shrinkage and the resulting reduction in intracranial pressure. These findings expand our understanding of antitumor mechanisms associated with LWF. However, clinical trials are needed to validate these findings. It has been shown that LWF has effective therapeutic effects in the malignant progression of glioma. It has significant advantages in preventing tumor recurrence, reducing toxic side effects, and prolonging survival.

## 4 Discussion

Glioma remains a focal point in oncology research. Modern therapies such as surgical resection, radiotherapy, and chemotherapy can moderately delay malignant progression. However, they offer limited long-term survival benefits. TCM demonstrates significant advantages in preventing tumor recurrence, reducing toxic side effects compared to conventional treatments, and prolonging survival. However, its complex mechanisms and bioactive components pose challenges for comprehensive elucidation.

In the past, technical compatibility only focused on addressing water retention, detoxification, and diarrhea. Our preferred ingredients for this formula are summer dry grass and dried tangerine peel, both of which have bitter and pungent rising powder. The ability to induce menstruation with one cold and one heat is doubled, which can directly penetrate the brain. Half branch lotus and white hedyotidis diffusae herba have been experimentally verified to have anti-cancer and anti-glioma effects. Bamboo stem and orange blossom red can cool and warm, which can reduce the sticky properties of glioma. Therefore, the components can coordinate and promote each other, playing a synergistic role and having good antitumor effects (Kubatka et al., 2025).

Through the glioma model experiments, clinical cases, network pharmacology analysis, SPR, and WB assay, we verified that LWF inhibited EGFR-induced glioma growth and suppressed EGFR, P-PI3K, PI3K, AKT, and P-AKT expression, to block the EGFR and PI3K–AKT pathways. In the network pharmacology analysis, we discovered a close connection between the two signaling pathways. Previous studies have also shown that the key downstream molecules of the EGFR signaling pathway include PI3K and AKT, which together form the PI3K–AKT signaling axis, playing pivotal roles in cell survival, proliferation, metabolism, and migration. LWF. Based on the above, we speculate that LWF can inhibit the PI3K signaling pathway by blocking EGFR, thereby suppressing cell proliferation and exerting antitumor effects.

Analysis of the pharmacodynamic mechanism and functional material basis of TCM is crucial for current research on LWF in treating glioma. To thoroughly investigate the material basis of LWF’s anti-glioblastoma effects, we employed network pharmacology, flow cytometry, SPR, and molecular docking. Analysis revealed that baicalin and luteolin can target EGFR, inhibit the EGFR/PI3K/AKT signaling pathway, and enhance T cell antitumor immunity to exert antitumor effects.

Regarding the antitumor effects of baicalin and luteolin, numerous researchers have reported significant findings. Wei Tao et al. discovered that baicalin from *Scutellaria barbata* downregulates FTH1 to induce ferroptosis in bladder cancer cells (Kong et al., 2021). Mengxian Li et al. found that baicalin induces apoptosis in lung cancer cells by regulating the glutamine-mTOR metabolic pathway (Li J. et al., 2025). Gang Huang et al. demonstrated that baicalin induces death in non-small cell lung cancer cells via MCOLN3-mediated lysosomal dysfunction and autophagy blockade (Dong et al., 2024). Wang Qilong et al. demonstrated that luteolin–paclitaxel composite bifunctional liposomes exert significant synergistic effects against esophageal squamous cell carcinoma while alleviating paclitaxel-induced hepatotoxicity (Sun et al., 2025). Pu Xiaohui et al. (Zong et al., 2025) developed carrier-free rod-shaped luteolin-glycyrrhizin acid self-assembled nanoparticles that exhibit dual-target synergistic activity against liver cancer. In summary, baicalin and luteolin show considerable promise as antitumor functional materials.

Despite these encouraging findings, several limitations should be acknowledged. This study identified baicalin and luteolin as the key active components in LWF that target EGFR through network pharmacology and molecular docking; however, these two individual compounds cannot fully represent the overall mechanism of action of the compound formula. To address this limitation, future research could be expanded in the following areas: First, analyze the components of LWF that enter the bloodstream and those that migrate into brain tissue. This would clarify the profile of components in the compound that can actually be absorbed into the bloodstream and cross the blood-brain barrier and verify the actual *in vivo* exposure levels of baicalin and luteolin. Second, conduct correlation analyses between the chemical fingerprint profiles and pharmacodynamic data of multiple batches of LWF to identify the groups of components closely associated with anti-glioma activity, without compromising the integrity of the compound.

We first proposed using both for the treatment of glioma by targeting EGFR, which provides a critical foundation for developing novel EGFR small-molecule inhibitors. Elucidating this bioactive basis is critical for identifying LWF’s anti-glioma constituents and accelerating clinical translation of EGFR-targeted glioma therapeutics. Further development of baicalin and luteolin combination therapy for glioma may yield clinical benefits in the future.

Integrating clinical analysis with network pharmacology and experimental validation, we identify EGFR/PI3K/AKT pathway modulation and enhanced T cell antitumor immunity as important anti-glioma mechanisms of LWF. Baicalin and luteolin were identified as two key bioactive constituents within LWF that contribute significantly to EGFR inhibition. However, it should be noted that the holistic effects of the multi-component LWF formula cannot be fully represented by these two monomers alone. Future studies are required to elucidate the synergistic actions of other components and their contributions to the overall therapeutic efficacy of LWF.

We also explicitly mention the limitations of this model in the discussion, as it fails to recapitulate the blood–brain barrier and the unique central nervous system immune microenvironment critical for evaluating glioma therapeutics. Nevertheless, certain aspects of the study require additional investigation to increase the robustness of the conclusions. We used a xenograft Luc-GL261 glioma mouse model, which could partially evaluate LWF’s efficacy, but it fails to duplicate the blood–brain barrier and the unique central nervous system immune microenvironment critical for evaluating glioma therapeutics.

Animal studies showed that the tumor-suppressing effect of low-dose LWF in combination with bevacizumab was significantly superior to that of either agent alone. However, there was no statistically significant difference between the high-dose LWF monotherapy group and the combination group (p > 0.05). This suggests that high-dose LWF alone is already approaching its maximum effect, and combination therapy did not provide additional benefits. Furthermore, the two drugs have different mechanisms of action: bevacizumab acts on tumor vasculature, while LWF targets the EGFR pathway in tumor cells and modulates the immune system. Their complementary effects provide a basis for clinical combination therapy.

## 5 Conclusion

Following confirmation of LWF’s efficacy against glioma progression via murine pharmacodynamic assays and H&E staining, network pharmacology screening identified core targets, including EGFR, PI3K, and AKT. Subsequent GO functional and KEGG pathway enrichment analyses revealed LWF’s anti-glioma mechanisms primarily involve EGFR- and PI3K-AKT-mediated pathways regulating cellular proliferation and apoptosis signaling, increasing the infiltration of CD8^+^T cells, and enhancing the antitumor immune response.

Prompted by these findings, *in vitro* cellular experiments validated the network pharmacology predictions: LWF significantly inhibited glioma progression by suppressing EGFR. Molecular docking confirmed these results, aligning with our initial hypotheses. Integrating clinical analysis with network pharmacology and experimental validation, we identify EGFR/PI3K/AKT pathway modulation, cytotoxic T cell infiltration, and T-cell antitumor immunity as LWF’s primary anti-glioma mechanisms.

## Data Availability

The original contributions presented in the study are included in the article/Supplementary Material, further inquiries can be directed to the corresponding authors.

## Supplementary material

SUPPLEMENT FIGURE S1 XIC of luteolin, wogonin, poncirin, and quercetin. **(A)** XIC of luteolin. **(B)** XIC of wogonin. **(C)** XIC of poncirin. **(D)** XIC of quercetin.

SUPPLEMENT FIGURE S2 The analysis results of LWF in Clinical Practice Liu. **(A)** Liu’s initial diagnosis report. **(B-D)** Liu’s diagnosis report after the administration of LWF.
