# Supplementary figures and images for: Liu Wei formula ameliorates EGFR-induced malignant glioma progression by regulating EGFR/PI3K/AKT pathway and T-cell antitumor immunity
Source: Front Pharmacol. 2026 Jul 13;17:1825282. doi: 10.3389/fphar.2026.1825282 (PMC13402800; doi:10.3389/fphar.2026.1825282)

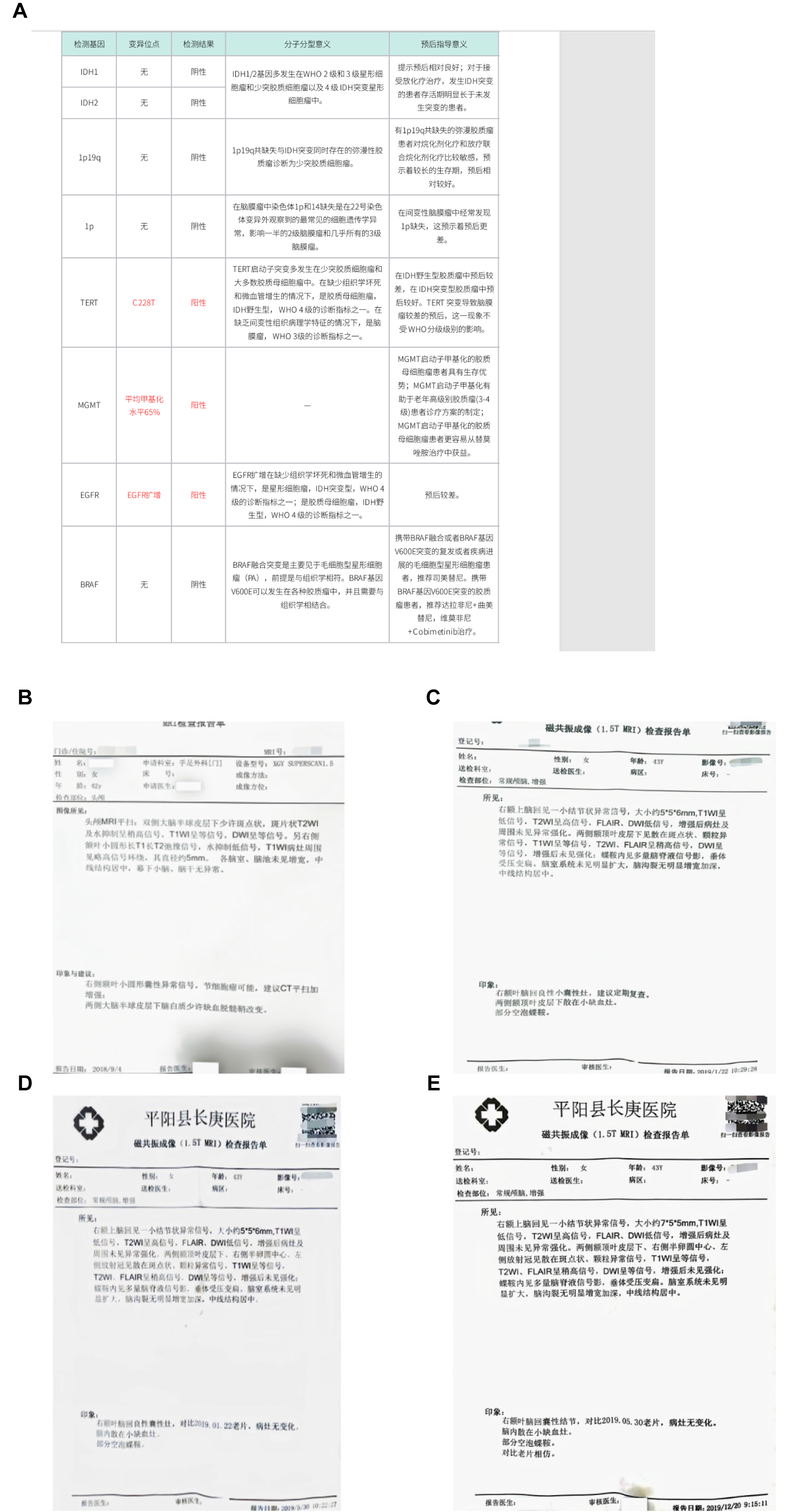

Supplement: Supplementary file 1 [file Image2.tif]

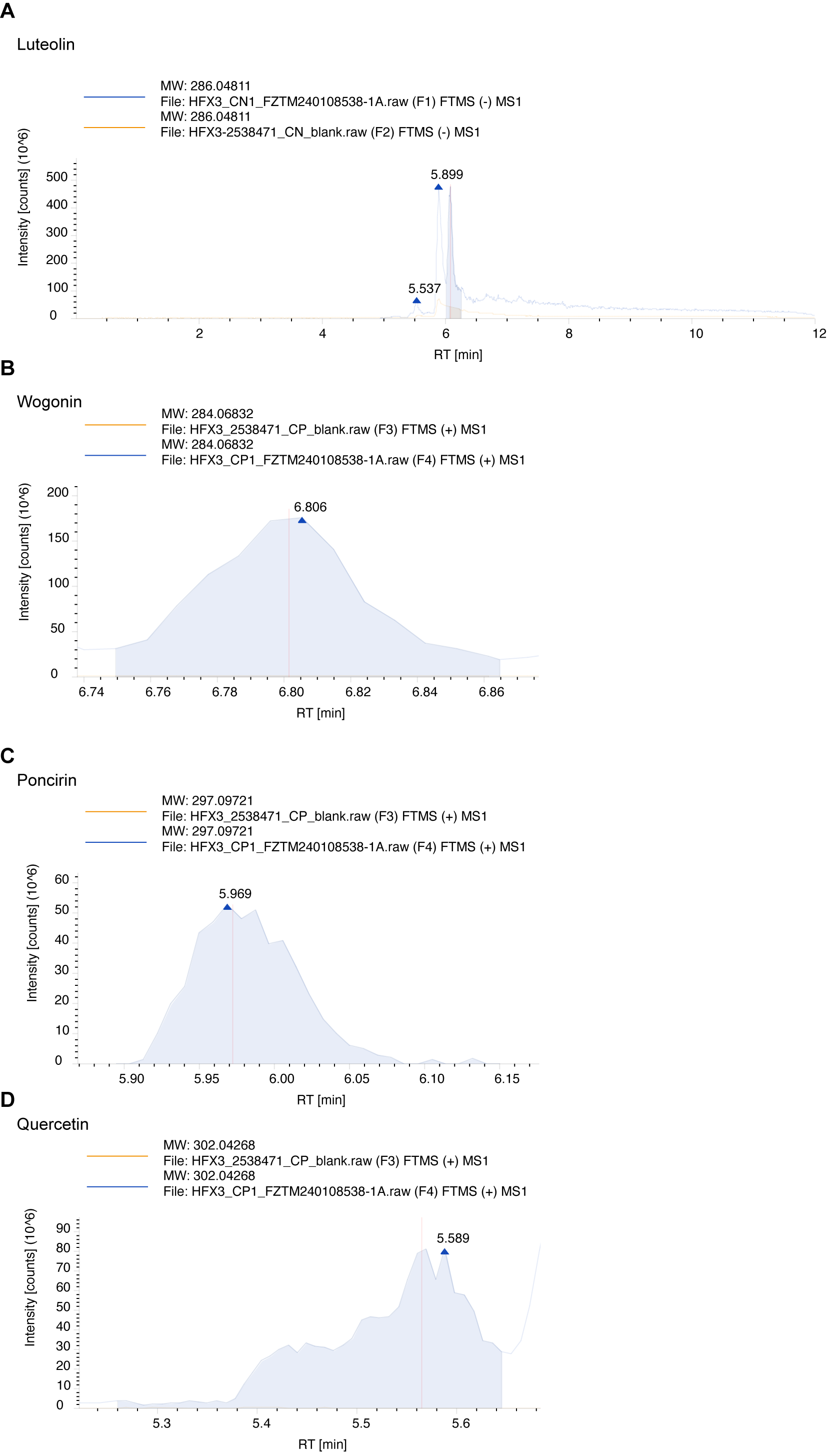

Supplement: Supplementary file 2 [file Image1.tif]
